# Design of a new multi-epitope peptide vaccine for non-small cell Lung cancer via vaccinology methods: an *in silico* study

**DOI:** 10.22099/mbrc.2022.42468.1697

**Published:** 2022-03

**Authors:** Fatemeh Heidary, Mehdi Tourani, Fatemeh Hejazi-Amiri, Seyyed Hossein Khatami, Navid Jamali, Mortaza Taheri-Anganeh

**Affiliations:** 1 Department of Mycology, Faculty of Medical Sciences, Tarbiat Modares University, Tehran, Iran; 2 Cellular and Molecular Biology Research Center, Health Research Institute, Babol University of Medical Sciences, Babol, Iran; 3 Department of Microbiology, Faculty of Medicine, Babol University of Medical Sciences, Babol, Iran; 4 Department of Clinical Biochemistry, School of Medicine, Shahid Beheshti University of Medical Sciences, Tehran, Iran; 5 Department of Laboratory Sciences, Sirjan School of Medical Sciences, Sirjan, Iran; 6 Cellular and Molecular Research Center, Cellular and Molecular Medicine Institute, Urmia University of Medical Sciences, Urmia, Iran; # Fatemeh Heidary and Mehdi Tourani are contributed equally to this work.

**Keywords:** Lung cancer, Bioinformatics, Epitope Vaccine

## Abstract

Lung cancer is the most common type of tumor worldwide. Non-small-cell lung carcinoma (NSCLC) is considered any epithelial cell-related lung cancer, which includes more than 85% of all lung cancer cases. NSCLC is less responsive to chemotherapy than SCLC. Therefore, the need for other treatments has become more pronounced and immunotherapy has gained increasing attention as a promising therapy in recent years. The current study aimed to design a multi-epitope peptide vaccine targeting main cancer/testis antigens of SP17, AKAP4, and PTTG1, which have a major function in tumor cell proliferation invasion. The protein vaccine was constructed using the rigorous immunoinformatics analysis and investigation of several immune system parameters, considering B cell epitopes and CD4 and CD8 induced epitopes as the most important cells to respond to cancer cells. Inverse translation and optimization of codons were performed to have the designed protein's cloning as well as expression potential in *E.coli.* Physicochemical, antigenic, and allergenic features were assessed to confirm the safety and immunogenicity of the vaccine. The secondary and tertiary structures were predicted. Finally, intrinsic disorder and 3D model refinement and validation were performed to eliminate structural problems. The designed construct had a stable structure that could be an antigen and stimulate the immune system and not be an allergen. The built model 3D structure was valid and stable. Further investigations are needed to approve the safety and immunogenic property of this new vaccine for NSCLC before it can be used in patients.

## INTRODUCTION

Cancer deaths from lung cancer have increased in recent years, but its prevalence varies considerably around the world [1]. In 2019, about 228,150 lung cancer new cases and 142,670 deaths related to lung cancer-associated death are estimated for the United States alone. Approximately 85% of lung cancer patients are attributed to non-small cell lung cancer (NSCLC), and has a 5-year overall survival rate of 23%, compared to only 6% for small cell lung cancer [2]. It is important to note that despite efforts and advancements in cancer diagnosis and treatment, many patients with NSCLC succumb to their disease. This life-threatening disease necessitates the development of more effective treatment options [3, 4]. Immunotherapy has been recently suggested as a promising treatment option for cancers such as NSCLC. Recently, the use of tumor-specific antigens and other effective protein sequences to stimulate the immune system and produce protective immune responses for those susceptible to or involved in cancer has been highly regarded [5-11]. Current evidence demonstrated that cancer testis antigens (CTA) such as pituitary tumor transforming gene 1 (PTTG1), A-kinase anchor protein 4 (AKAP4), and sperm protein 17 (SP17) are potential immunotherapeutic targets in NSCLC [12, 13]. The importance of CTAs, a class of tumor-associated antigens, is due to their high expression in cancerous tissues and their rarity in normal ones [14].

For vaccine design and development, multiple-epitope subunit vaccines are favored in comparison with single epitope ones as (I) they contain major histocompatibility complex (MHC)-restricted epitopes which could be identified by T cell receptors of diverse subsets of T-cells; (II) they possess B-cell, T helper and cytotoxic T lymphocytes epitopes which can trigger robust cellular and humoral immunity concurrently; (III) they comprise several epitopes of variant virus or tumor antigens which can extend to a wide range of targeted viruses or tumors, (IV) they present several components with adjuvant property which can boost the strength and duration of immune responses; and (V) they decrease undesirable components that can induce damaging immune response or adverse side effects. Therefore, such well-designed multiple-epitope subunit vaccines with several benefits can be potent therapeutic and prophylactic agents against cancers [15].

Identification of immunogenic CTA peptides and their application in multi-epitope vaccines have shown promising antitumor properties. Functional confirmation tests of these vaccines can be verified first in the bioinformatics phase and later in the *in vitro* and *in vivo* stages. To date, no comprehensive studies have been performed on the SP17, AKAP4, and PTTG1 antigens to identify their best epitopes [13, 16]. 

In addition to the employed antigens, various strategies can be utilized to increase the immunogenicity of the designed vaccines [17-19]. One of these strategies is the addition of helper T cell-stimulating epitopes as a pivotal cell in controlling the immune system network. These epitopes are found in some bacterial toxins such as TTFrC (tetanus toxin fragment c), cholera toxin, and LT (heat-labile enterotoxin) and help to overcome the strategy of cancer cells [20]. The ability of the immune system to specifically inhibit cancer cells makes it the most potent weapon for long-term tumor control. Multi-epitope vaccines have many advantages, including high specificity, stability under different conditions according to predetermined studies, high immunogenicity along with minimal side effects (due to controlled immune stimulation), and their cost-effective production [21].

Given the advances in bioinformatics in the last decade, it is possible to identify the most immunogenic epitopes using algorithms in biological databanks. These investigations appear to be necessary before experimental studies [20]. Keeping in view these data, the present study aimed to devise a multi-epitope subunit vaccine targeting SP17, AKAP4, and PTTG1 in NSCLC. In this regard, we have also designed a protein construct and determined its physicochemical and immunogenic properties using online tools. Several immune-related indexes evaluated its recombinant production capacity.

## MATERIALS AND METHODS

**Collection of data and protein sequences:** The amino acid sequence of SP17 (Accession no. Q15506), AKAP4 (Accession no. Q5JQC9), and PTTG1 (Accession no. O95997) were retrieved and saved in FASTA format from the National Center for Biotechnology Information (NCBI) and the Universal Protein Resource (UniProt) at http://www.uniprot.org. Multiple sequence alignments (MSAs) were carried out using sequences stored in the NCBI database to determine the sequence similarity (http://www.ncbi.nlm.nih/Blast).

**Immuno-informatics analyses: **Epitope prediction major histocompatibility complexes I and II: After the first encounter of antigens with antigen-presenting cells (APCs), MHC molecules present epitopes of antigens to the T cells. Therefore, antigen evaluation in terms of MHC identification is a crucial early stage in vaccine design. A variety of analytical algorithms can predict MHC class I-binding peptide sequences. The NHLAPred, a neural network-assisted prediction server for MHC class-I binding peptide, was firstly used (https://webs.iiitd.edu.in/ tmp/nhlapred01.html). Immune Epitope Database Analysis Resource (IEDB) online server [22]. was used to provide a quantitative matrix for the broad range alleles of the MHC Class-I to forecast the best epitopes. RANKPEP online software was also utilized to predict epitopes of MHC class II for all three chosen proteins. Epitopes which were bound to the maximum number of MHC alleles were appointed, especially those alleles involved in the detection or protection of NSCLC.

**Cytotoxic T lymphocyte epitopes prediction** **: **An online server called CTLpred was chosen to forecast the epitopes which could stimulate T cells in the preparation of a direct method (http://crdd.osdd.net/raghava/ctlpred/). This online server makes this prediction using information and models of T cell-stimulating epitopes (regardless of MHC molecule presentation). This method depends on learning machine algorithms such as artificial neural network (ANN) and support vector machine (SVM) [14].

**B-cell epitopes Prediction: **B-cell receptors can detect linear as well as conformational epitopes of antigens. The IEDB server (http://tools.iedb.org/bcell/) was used for the prediction of linear epitopes of B cell. The Discotope server (http://www.cbs.dtu.dk/services/DiscoTope/) was applied to identify Continued epitopes of B cell on 3D protein structures. Epitope surface accessibility and epitope affinity are important factors in selecting the best conformational epitopes [23, 24].

**Chimeric gene design and optimization: **Appropriate sequences of SP17, AKAP4, and PTTG1 antigens were selected to determine the chimeric structure to stimulate T and B cells. In addition, TTFrC was used as an epitope to produce helper T cells and heparin-binding hemagglutinin as an adjuvant. The selected peptide fragments were ligated to each other by suitable EAAAK, GPGPG, and HEYGAEALERAG linkers. Different servers evaluated the performance characteristics of the compiled structure. It is necessary to investigate its compatibility with *E.coli* codons using inverse translation and optimization of codon before protein construction to clone and express the designed protein in the *E. coli* host. For this purpose, JCAT software (http://www.jcat.de) was used before and after refinement. The Vaxigen server also predicted the immunogenicity of the chimeric protein and its subunits.

**Vaccine features: **Evaluating physicochemical parameters and assessment of allergenicity: Theoretical pI (isoelectric point), Grand averages of hydropathicity (GRAVY), molecular weight, , aliphatic index, half-life, instability index, and amino acid composition were assessed using the ProtParam server23 (http://web.expasy.org/protparam) to determine the physicochemical features. It is necessary to evaluate the status of allergenicity and antigenicity. The construct can make the immune system hypersensitive or insensitive. For this purpose, AlgPred web server (http://www.imtech.res.in/raghava/algpred) was utilized to test these indexes based on six different methods with an accuracy of 85% and a threshold of -0.4. 

**Prediction of secondary and tertiary structure of multi-epitope vaccine:** It was necessary to use PDBsum and PSIPRED for secondary structure prediction and further definition of the structural characteristics of the designed construct. PDBsum can be used for the identification of the molecule(s) which are responsible for constructing the structures of ligands, DNA, proteins, and metal ions [25]. In addition, it shows the ligand-protein and individual chain interactions as well as protein secondary structure. Reported data also contains complete information about structural motifs such as channels, pores, and apertures. The tertiary structure of the multi-epitope subunit vaccine was forecasted using I-TASSER15 and Phyre2 online servers. These tools provide hierarchical methods for predicting protein structure and function [26, 27]. 

**Validation and Refinement of the 3D modeled structure:** The GalaxyRefine online server (http://galaxy.seoklab.org/cgi-bin/submit.cgi?type=REFINE) was applied to enhance the quality of the template-based constructed model and create a more efficient form. The process uses both mild and aggressive relaxation methods to modify the protein. Operation strategy of the server for prediction of the vaccine 3D structure is based on the similarity status between the available template structure option and the target protein. Further structural relaxation and molecular dynamics simulation were shown by GalaxyRefine. Before and after the refinement process, a Ramachandran plot was made to verify the design using an online webserver called RAMPAGE (http://mordred.bioc.cam.ac.uk/~rapper/rampage.php). The PROSA service was used to perform additional protein structure validation. This system can be used to calculate the proposed structure's overall quality score. Notably, if the score for the sequences falls outside the specified range, the structure is likely to contain errors.

**Predicting inherent protein disorder: **Given the fact that the construct is not native, there may be some defects. So, DisEMBL 1.5 (http://dis.embl.de/) and IUPred (http://iupred.enzim. hu/pred.php) servers were used to predict areas with defective structure and inherent protein disorders.

## RESULTS

The total number of amino acids in the final protein sequence was 364. As the construct's first domain, HBHA was chosen as an adjuvant in the N terminus. As the second domain, two regions from TTFrC were used as the epitopes of CD4+ helper (See appendix Table 1). The third domain was considered based on CTL epitopes selected from SP17, AKAP4, and PTTG1 protein sequences. Ultimately, valuable linkers were applied to link the favorite peptide sequences (See appendix Fig. 1).

The high-scored peptide regions over mouse-related MHC-I prevalent alleles (MHC-2Db, MHC-2Dd, MHC-2Kd) were selected from IEDB and NHLAPred online servers for each of three NSCLC-related antigens (sp17, AKAP4, and PTTG1) (Tables 1, 2, and 3). Finally, two areas from SP17 (between 29–43 and 118-145 amino acid residues), three areas from AKAP4 (between 18-27, 206-215, and 213-222 amino acid residues) and three regions from PTTG1(between 40-49, 27-36, and 159-168 amino acid residues) were selected for analysis. 

**Table 1:** TTFrC MHC-II binding peptides determined by RANKPEP

**Epitope sequence**	**Start position**	**End position**
NDIISDISGFNSSVITYPDAQLVPGINGKAIHLVNNE	40	66
IEYNDMFNNFTVSFWLRVPKVSASLEQYGT	78	108

**Table 2:** SP17 MHCI and II predicted epitopes

**Server**	**HLA Class-I**	**Start**	**End**	**Sequence From CD81**	**Best ranked epitope**
** Low percentile rank = good binders**					**Percentile rank**
**IEDB**	H-2-Db	34	43	NIPAFAAAYF	1.4
	H-2-Dd	38	47	FAAAYFESLL	2.75
	H-2-Kb	37	46	AFAAAYFESL	2.85
** high score= good binders**					**SCORE**
**NHLAPred**	H2_Db	34	43	NIPAFAAAYF	20.364
	H2_Dd	30	39	EQPDNIPAF	18.510
	H2_Kb	39	48	AAAYFESLL	15.201
	HLA Class- II	**high score= good binders**			Core reliability score
**IEDB**		113	127	KEEVAAVKIQAAFRG	0.06
		29	43	REQPDNIPAFAAAYF	1.15
**RANKPEP**	** high score= good binders**				**SCORE**
	H-2-IAd	29	38	REQPDNIPA	11.704
	H-2-IAb	116	125	VAAVKIQAA	11.24

**Table 3:** AKAP4 MHCI and II predicted epitopes

**Server**	**HLA Class-I**	**Start**	**End**	**Sequence From CD81**	**Best ranked epitope**
	** Low percentile rank=good binders**				**Percentile rank**
**IEDB**	H-2-Db	159	168	YADQVNIDYL	0.16
	H-2-Dd	18	27	RSHRGVCKV	0.23
	H-2-Kb	218	227	SFYVNRLSSL	0.32
	** high score=good binders**				**SCORE**
**NHLAPred**	H2_Db	206	215	ISPDGECSI	22.365
	H2_Dd	213	222	SIDDLSFYV	20.369
	H2_Kb	22	31	KSQSLSYASL	18.325
	**HLA Class-II**	** high score=good binders**			**Core reliability score**
**IEDB**	H-2-IAd	221	235	VNRLSSLVIQMAHKE	0.68
	H-2-IAb	205	220	VISPDGECSIDDLSF	0.51
**RANKPEP**	** high score=good binders**				**SCORE**
	H-2-IAd	205	214	VISPDGECS	6.678
	H-2-IAb	736	745	FRGTRCIHS	5.255

The most important part of the immune system that needs to be stimulated in cancer vaccine production is cytotoxic T cells. Therefore, selecting the most accessible and stimulating epitopes that antigen-presenting molecules have previously confirmed is important. Accordingly, the CTLPred online server (http://crdd.osdd.net/raghava/ctlpred/) found that the best presentable epitopes on MHCs overlap with T cell epitopes on sp17, AKAP4, and PTTG1. These epitopes were mentioned above. 

CTLPred uses a direct method relying on the information and pattern of T-cell epitopes regardless of MHC responses, and through an artificial neural network [14] and support vector machine (SVM) to predict CTL epitopes. 

The appropriate amino acid regions for presentation by MHC-II were also selected by IMMUNE EPITOPES DATABASE (IEDB) and RANKPEP server, and the best epitopes presentable by these two vital molecules were chosen for identification by T cells. Various prediction methods used by the IEDB server, which results in choosing the best immunogenic molecules. The server employs the Consensus, Combining NN-align, SMM-align, and CombLib methods. If these methods fail to predict the appropriate epitopes, the alternative way NetMHCIIpan will be used. Given that the designed vaccine ultimately needs to be functionally evaluated in mouse models, the common mouse alleles H2-IAb and H2-IAd were selected (Table 4).

BCPred software was applied to forecast the continuous B-cell epitopes. All 16-mers with BCPred cutoff score >0.9 in epitopes of B-cell were chosen (Table 5). 

**Table 4:** PTTG1 MHCI and II predicted epitopes

**Server**	**HLA Class-I**	**Start**	**End**	**Sequence From CD81**	**Best ranked epitope**
	** Low percentile rank=good binders**				**Percentile rank**
**IEDB**	H-2-Db	42	51	STPRFGKTFD	2.2
	H-2-Dd	170	179	PSPPWESNLL	1.15
	H-2-Kb	114	123	IEKFFPFNPL	1.3
	** high score=good binders**				**SCORE**
**NHLAPred**	H2_Db	40	49	QVSTPRFGK	25.366
	H2_Dd	27	36	GSGPSIKAL	21.324
	H2_Kb	159	168	FQLGPPSPV	22.326
	**HLA Class-II**	** high score=good binders**			**Core reliability score**
**IEDB**	H-2-IAd	155	169	LEKLFQLGPPSPVKM	0.83
	H-2-IAb	46	60	FGKTFDAPPALPKAT	0.93
**RANKPEP**	** high score=good binders**				**SCORE**
	H-2-IAd	50	59	FDAPPALPK	11.84
	H-2-IAb	158	167	LFQLGPPSP	5.289

**Table 5:** Predicted T cell epitopes using different servers

**Peptide rank**	**Start position**	**sequence**	**Score(ANN/SVM)**
** SP17**			
**1**	35	IPAFAAAYF	0.97/0.92642045
**2**	118	AVKIQAAFR	0.94/0.46358695
**3**	30	EQPDNIPAF	0.58/0.72258157
			
** AKAP4**			
**1**	18	RSHRGVCKV	0.98/0.98838417
**2**	206	ISPDGECSI	0.80/0.91083083
**3**	213	SIDDLSFYV	0.92/0.77818421
			
** PTTG1**			
**1**	40	QVSTPRFGK	0.92/0.68594268
**2**	27	GSGPSIKAL	0.97/0.4606087
**3**	159	FQLGPPSPV	0.96/0.45444044

The continuous B-cell epitopes used in this study were chosen based on a variety of factors such as antigenicity, hydrophilicity, polarity, accessibility, flexibility, and exposed surface area. Obtained results indicated that no epitope was found at the linker sites among distinct domains (amino acids 501 to 505). Predicting discontinuous B cell epitopes was accomplished by the Discotope server, which identified B cell epitope residues out of 364 residues (See appendix Table 2).

The sequence of the multi-epitope vaccine begins with heparin-binding hemagglutinin adhesin, a critical virulence factor of *mycobacterium tuberculosis*, as an adjuvant in the N terminus. The protein is located on the bacterial cell surface, acts as a molecule adhering to non-phagocytic cells, and has a function in the extrapulmonary spread of the bacterium. In the following, two regions from TTFrC were used to more stimulate the helper T lymphocyte responses. Epitopes chosen from SP17, AKAP4, and PTTG1 protein sequences were added as the central part of the vaccine against NSCLC to intensify CTL-related immunity reaction. Appropriate linkers are required to maintain the 3D functional structure of the selected sequences in the designed vaccine. The best of these GPGPG and EAAAK were used.

As mentioned above, the final sequence of vaccine consists of 364 amino acids and three domains. JCat prepares the inverse translation and optimization of codon. Codon adaptation index (CAI) was obtained 0.81 (See appendix Fig. 2). Increased expression level in the host required a CAI of more than 0.8. To put it another way, the gene sequence's CAI is 0.85, which means it's suitable for high-level expression in this particular host. 55.42 percent of the total GC content was reported; the optimal GC content range is considered to be between 30 and 70 percent. Only one cis-acting negative element was found in the gene.

The calculated molecular weight and isoelectric point (pI) were found to be 39.517 and 4.87 for the chimeric proteins. At 280 nm, the chimeric protein had an extinction coefficient of 23505 M^-1^ cm^-1^. While the half-life in mammalian and yeast reticulocytes was estimated at 30 hours, it was over 20 hours in *E.coli* and yeast. It has a stability index of 37.19 and is considered as a stable protein according to Expasy ProtParam. Aliphatic index and GRAVY of chimeric proteins were defined as 84.07 and -0.334, respectively. Allergenic proteins and IgE epitopes were predicted and mapped. However, based on amino acid composition, there were no IgE epitopes in the protein sequence, and the protein was found to be nonallergenic (See appendix Table 3).

**Figure 1:**
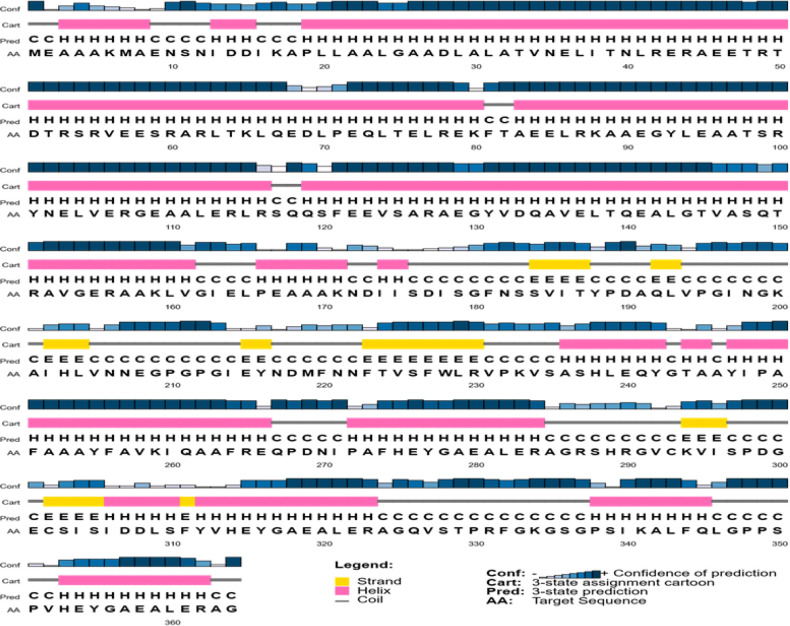
Graphical results for secondary structure prediction of chimeric protein

**Figure 2:**
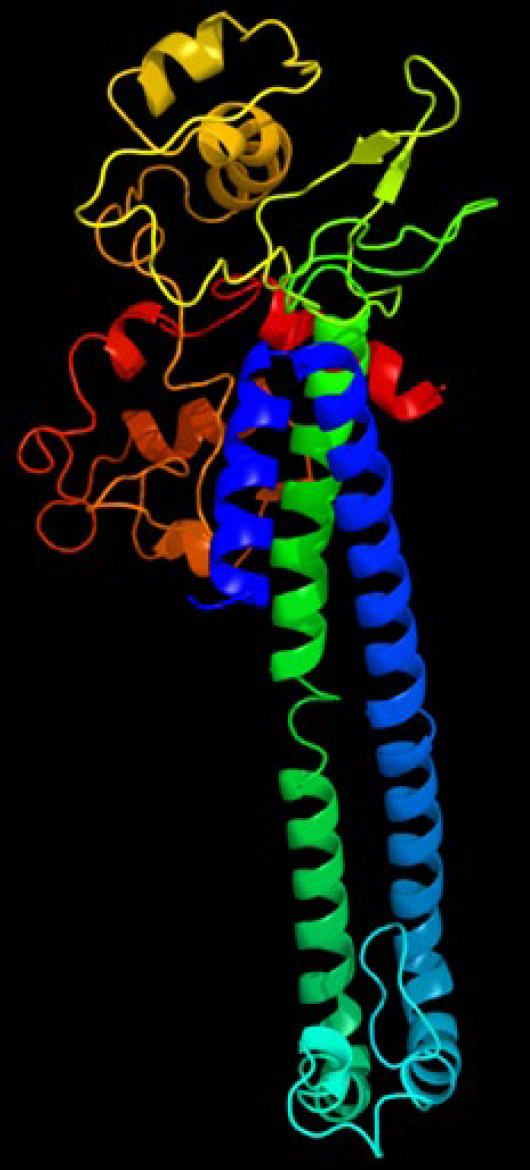
Predicted structure of constructed protein using I-TASSER and Phyre2 software. The three-dimensional structure showed a protein with three main domains linked together with appropriated linker

**Figure 3:**
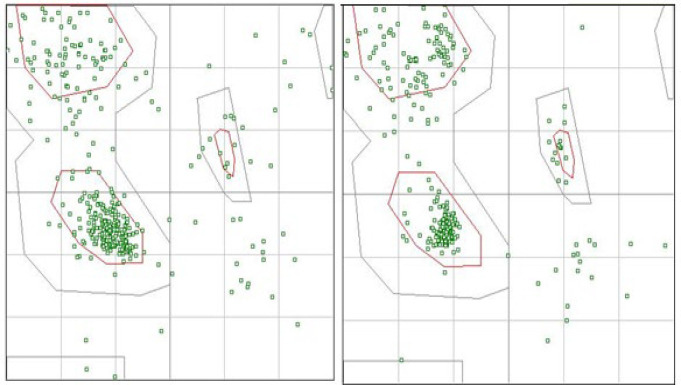
Validation of protein 3D model, before and after refinement by Ramachandran plot. (A) In initial model, 276 (76.2%), 48 (13.3%) and 38 (10.5%) of residues were located in favored, allowed and outlier regions, respectively. (B) In refined model, in refined model, 326 (90.1%), 26 (7.2%) and 10 (2.8%) of residues were located in favored, allowed and outlier regions, respectively

Online server predicting the secondary structure of the chimeric protein is given in Figure 1. It was discovered through the research that the protein's structural contents include alpha helix, a random coil, and extended strands. Furthermore, the chimeric proteins' predicted secondary structure included 62-64 % alpha-helix, 8.79 % extended strands, and 28.57 % random coil. The I-TASSER and Phyre2 online server programs were applied for the 3D structure of the chimeric protein. Three main domains were found to be linked by a linker in a protein discovered by the I-TASSER method (Fig. 2). For the I-TASSER-predicted models, the C-score was a -1.10. In most cases, the C-score is in the -5 range, and the higher the C-score value, the more confident the model. Also included in this model were the template modeling (TM) score and the root-mean-square deviation (RMSD), both of which were 0.580.14 and 9.14.6, respectively. The Ramachandran plot was used to analyze the chimeric protein. According to the results, the vast majority of the amino acid residues of modeled structure were included into the plot's allowed and favored categories (Fig. 3).

**Figure 4:**
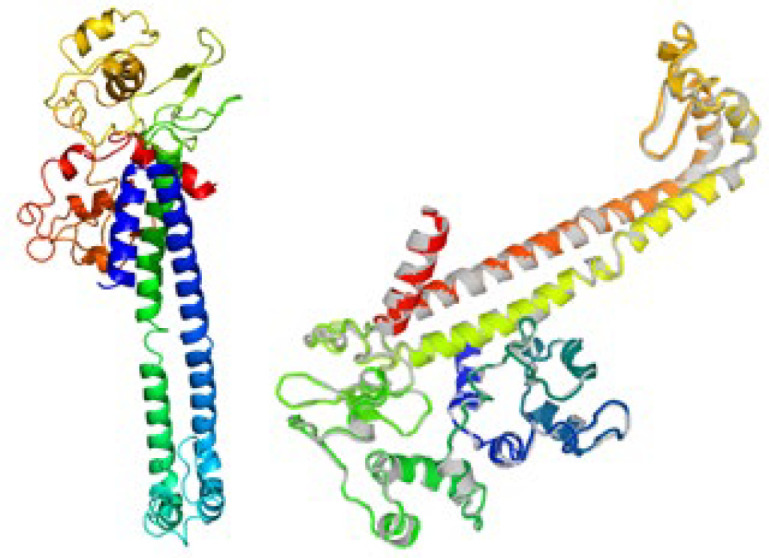
Tertiary structure of modeled vaccine before refinement (Left). Superimposition of tertiary structure of modeled vaccine after refinement (Right).

To refine the acquired tertiary structures, the GalaxyRefine program was used. Subsequently, the refined 3d structures were validated by ProSA-web server and Ramachandran Plot (Fig 3). Using the Ramachandran plot, protein validation of the 3D model indicated that 276 (76.2%), 48 (13.3%), and 38 (10.5%) of residues were placed in the favor, allowed and outlier categories of initial model, respectively. In the Refinement model, 326 (90.1%), 26 (7.2%), and 10 (2.8%) of residues were placed in the allowed, favored, and outlier categories, respectively (Fig. 4).

**Figure 5:**
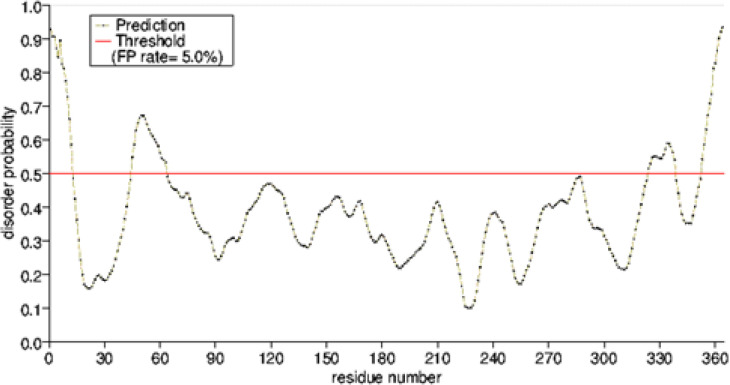
Intrinsically disorder regions. Amino acids in the input sequence were considered disordered when the black line is above the red line

As noted, the disordered areas were identified by DisEMBL online software. Amino acids 2-12, 45-60, 320-342, and 355-361 were considered disordered areas, considering Loops/coils, hot-loops, and the remark-465 definition (Fig. 5). 

## DISCUSSION

In recent years, immunotherapy has risen to prominence as a promising cancer treatment and prevention method. Cancer treatment relies heavily on the induction of antigen-specific immune responses and/or T-cell checkpoint modulation to enhance anti-tumor activity. [28]. Nowadays, bioinformatics changes biology and medicine and opens new horizons in designing novel molecules and processes in biomedicine fields [29-34].

Therefore, in the current study, we attempted to devise a multi-epitope-based vaccine via bioinformatics tools targeting the major tumor-associated antigens including SP17, AKAP4, and PTTG1, which have been implicated in in tumor proliferation and invasion. Since different alignments of amino acid sequences together can lead to different spatial protein structures, particular epitopes must be properly sequenced to produce a functional vaccine [20, 35]. 

HBHA in the N terminus was chosen as a first domain and an adjuvant for the construct. Two regions of TTFrC were used as the epitopes of CD4+ helper in the second domain. The third domain was selected from SP17, AKAP4, and PTTG1 protein sequences as CTL epitopes. To connect favorite peptide sequences, rigid linkers were eventually used. The constructed proteins required an appropriate linker to bind different favorite epitopes. It is imperative that we effectively detach the domains of the desired chimeric protein using GPGPG, EAAAK, and HEYGAEALERAG cleavable linkers. The findings of bioinformatics and our successful use of chimeric gene's linkers have demonstrated their usefulness in the construction of functional structures [36]. 

The selected linker can notably regulate the distance between epitopes and decrease the interference between the domains [37]. ProtParam software was used to analyze the physicochemical parameters of our chimeric sequence. The pI value (pI>7.75) indicated the protein's fundamental nature. At 280 nm, the constructed protein had a high extinction coefficient. Expasy ProtParam tool categorized the studied chimeric protein as stable based on its instability index (35.59). Cellular immunity, as the most important part of the immune system against viral and cancer cells, relies on the activity of CTL cells. Therefore, the strategy of designing anticancer vaccines should be based on selecting the best T cell-stimulating antigens and presentable epitopes by MHCs, and then investigating the defects and potentials of the selected sequences to serve as ideal antigens [38]. The CTLpred online database was applied to identify potentially T-cell epitopes in chimeric proteins. Epitopes that were antigenic and could potentially interact with human HLA alleles were chosen. The prediction of the 3D structure of studied chimeric protein was done by I-TASSER software from the beginning. Results showed that the ab initio I-TASSER software predicted the folds for our multivalent protein and provided a good resolution model. It is possible to determine how reliable and accurate a predicted model is by checking the RMSD and TM scores [39]. 

According to the model, the accuracy is confirmed by the expected TM score of 0.580.14. ATM-score greater than 0.5 typically shows the accurate topology model. Also, the C- and Z-scores indicate the level of confidence in the topology model. Finally, the Ramachandran diagram analysis revealed a protein with complete stability [40]. From an applied aspect, a functional and effective constructed cancer vaccine should involve cellular and humoral immunity to overcome the growth rate and metastasis of cancer cells by utilizing all immune system's capacity, especially in the lung, as a vital tissue [41].

The chimeric protein's B-cell epitopes were discovered in this study by using a variety of different indexes, including antigenicity, hydrophobicity, accessibility, flexibility, and secondary structure analysis, among others. There were acceptable results from the assessment of all continuous B-cell epitopes in this study.

The initial function of B cells is related to the development of humoral immunity. In lung cancer, tumor-associated B cells affected by tumor antigen uptake differentiate into plasma cells, produce tumor-specific antibodies, and generate memory B-cells, which last in the involved host and lead to long-lasting responses against cancer antigens. There is a direct relationship between the severity of B cell response and the aggressiveness of cancer cells [42]. DiscoTope has proven that it is one of the most reliable software to recognize conformational B cell epitopes. Antibodies could easily bind to the protein's surface epitopes. In computational studies, another important step is the identification of discontinuous epitopes which are required for the interaction of antibody and antigen [43]. 

## Conflict of Interest:

All authors declare that they have no conflict of interest.
